# Risk of Depression in the Offspring of Parents with Depression: The Role of Emotion Regulation, Cognitive Style, Parenting and Life Events

**DOI:** 10.1007/s10578-019-00930-4

**Published:** 2019-11-05

**Authors:** Johanna Loechner, Anca Sfärlea, Kornelija Starman, Frans Oort, Laura Asperud Thomsen, Gerd Schulte-Körne, Belinda Platt

**Affiliations:** 1 Department of Child and Adolescent Psychiatry, Psychosomatics and Psychotherapy, University Hospital, Ludwig-Maximilian-University Munich, Nussbaumstraße 5a, 80336 Munich, Germany; 2 Department of Clinical Psychology and Psychotherapy, Ludwig-Maximilian-University, Munich, Leopoldstraße 13, 80303 Munich, Germany; 3 Research Institute of Child Development and Education, Faculty of Social and Behavioural Sciences, University of Amsterdam, Amsterdam, The Netherlands

**Keywords:** Depression, Offspring of parents with depression, Transmission of depression, Risk factors, Development of depression, Mediation, Prevention

## Abstract

Children of depressed parents are at heightened risk for developing depression, yet relatively little is known about the specific mechanisms responsible. Since preventive interventions for this risk group show small effects which diminish overtime, it is crucial to uncover the key risk factors for depression. This study compared various potential mechanisms in children of depressed (high-risk; n = 74) versus non-depressed (low-risk; n = 37) parents and explored mediators of parental depression and risk in offspring. A German sample of N = 111 boys and girls aged 8 to 17 years were compared regarding children’s (i) symptoms of depression and general psychopathology, (ii) emotion regulation strategies, (iii) attributional style, (iv) perceived parenting style and (v) life events. Children in the high-risk group showed significantly more symptoms of depression and general psychopathology, less adaptive emotion regulation strategies, fewer positive life events and fewer positive parenting strategies in comparison with the low-risk group. Group differences in positive and negative attributional style were small and not statistically significant in a MANOVA test. Maladaptive emotion regulation strategies and negative life events were identified as partial mediators of the association between parental depression and children’s risk of depression. The study highlights the elevated risk of depression in children of depressed parents and provides empirical support for existing models of the mechanisms underlying transmission. Interestingly, the high-risk group was characterised by a lack of protective rather than increased vulnerability factors. These results are crucial for developing more effective preventive interventions for this high-risk population.

**Electronic supplementary material:**

The online version of this article (10.1007/s10578-019-00930-4) contains supplementary material, which is available to authorized users.

## Introduction

### Children of Parents with Depression

Depression is one of the most common psychiatric illness causing great personal and economic burden for individuals, families, and society [1, 2]. One of the biggest risk factors for developing depression is having a parent suffering from depression [3, 4]. Offspring of depressed parents are three to four times more likely to develop a depressive disorder than children of non-depressed parents [5] and familial depression even has an impact on the third generation-affecting 59.2% of grandchildren (mean age 12 years) [6]. In addition, children of depressed parents are more likely to experience more severe and continuous courses of depression [7]. Nevertheless, Collishaw et al. [8] identified one-fifth of the offspring of parents with depression to be in good mental health. Consequently, identifying the potential mechanisms underlying this risk is crucial in order to increase resilience in children at risk.

The predominant model of the transgenerational transmission of depression by Goodman and Gotlib [9] proposes four mechanisms; (a) heritability, (b) innate dysfunctional neuro-regulatory factors, (c) negative maternal cognitions, behaviors, and affect, and (d) stressful context of the children’s life, which by leading to cognitive, affective and behavioral deficits in children convey elevated risk for depression. The current study investigates the nature of cognitive and affective (emotion regulation) deficits in children of depressed parents as well as the role of exposure to stressful life events and parenting style, since these factors are modifiable and have been the target of recent preventive interventions for children of parents with depression [10]. Readers are referred elsewhere to studies of the genetic heritability of depression [11–13] and the role of innate dysfunctional neuro-regulatory factors [14].

### Emotion Regulation

Deficits in emotion regulation strategies are associated with the development of youth depression (see Schäfer et al. [15], for a meta-analysis). Emotion regulation strategies are commonly distinguished as (1) adaptive (e.g. cognitive re-appraisal, problem solving, acceptance, distraction) or (2) maladaptive (e.g. rumination, suppression). These styles develop early and may be impaired in children with a depressed parent [16, 17]. Consequently, dysfunctional emotion regulation might be a crucial factor in the transmission of depression. Indeed, there are findings confirming associations between children’s and parents’ maladaptive emotion regulation (e.g. decreased levels of cognitive reappraisal) and children’s internalising symptoms in a community sample of parent–child dyads [18] and within samples of children of parents with depression [19–22]. Just one study has compared emotion regulation strategies between children of depressed versus non-depressed parents [23]. In this study the offspring (daughters) of mothers with depression were characterised by a more passive style of emotion regulation—less frequently engaging in adaptive strategies such as distraction.^1^ However, the findings of the study are only generalizable to young children (age 4–7 years) of depressed mothers: as far as we are aware no study has examined emotion regulation strategies in older children of depressed versus non-depressed mothers and fathers.

### Cognitive Factors

Cognitive factors (i.e. negative self-evaluation, a pessimistic world view, and hopelessness regarding the future) play a key role in the development of depression in adults [24] and adolescents [25–27]. Experimental studies have found children of depressed versus non-depressed parents to show cognitive vulnerabilities in the form of negative attention [28, 29] and interpretation [30, 31] biases, as well as less positive self-concept [31]. Of the two studies comparing memory biases between children at high and low risk for depression, one found children of depressed mothers to show a negative memory bias [32], whereas the other found no group differences [33]. Cognitive vulnerabilities such as hopelessness, pessimism and low-self-worth have even been observed in children of depressed versus non-depressed mothers as young as 5-years old [34]. The authors discuss whether higher rates of maternal criticism caused this cognitive vulnerability or whether children with this predisposition elicit more parental criticism. Other studies demonstrate that the cognitive style of children of depressed parents is correlated with both primary (changing the source of stress) and secondary (adapting to stressor, e.g. acceptance) coping strategies [20] as well as maternal affect [35]. Although these findings highlight the role of cognitive factors in the transmission of depression, there are few studies directly comparing the presence of more diverse cognitive vulnerabilities (e.g. attributional style) in children and adolescents of parents with and without a history of depression and how these might be linked to the development of depression in offspring.

### Parenting Style

Parenting style [36] and parent–child interaction are typically negatively affected by depression (see Lovejoy et al. [37] for a meta-analysis), being characterised by adverse parenting skills (being inconsistent, intrusive) and negative child outcomes (emotional maltreatment, irritability) [38, 39]. Parent–child interactions not only affect attachment style and resulting emotion regulation in children in the short-term [16, 17, 40], but have also been shown to have an impact on the child’s continuing psychological development [41, 42]. Inconsistent parenting can lead to unpredictable situations and feelings of confusion and insecurity in children of depressed parents, especially since offspring are mostly uninformed about their parent’s depression [43]. In addition, this negative parenting style is mirrored in negative interactions in conflict situations with their children that, in turn, were found to be associated with higher self-reported symptoms of depression [44] in youth. Whereas previous studies have mainly focused on self-reported or observed (maternal) parenting of younger children, fewer have investigated children’s perception of their parents’ behaviour in older children and adolescents.

### Stressful Life Events

Diathesis-stress models of youth depression posit that cognitive and affective vulnerabilities trigger a depressive episode when the young person encounters a stressful life event [45]. The offspring of depressed parents (compared to healthy controls) may be more likely to experience negative life events, due to environmental circumstances that might have played a role in the development of the parental depression in the first place such as financial problems or marital discord [46, 47]. Although stressful life events were found to mediate the relation between maternal and child depression in a community sample [48], another study found that the increased risk of depression in first-degree relatives of depressed patients could not be explained by an increase in negative life events [49]. One additional factor which may explain the discrepancy is that previous studies have relied on the raw number of life events experienced, rather than taking into account the subjective perception of stress associated with them.

### Summary and Current Study

Children of depressed parents have an increased risk of developing depression [5], which might be due to deficits in emotion regulation, cognitive vulnerability, negative parenting style, and stressful life events [9]. There is a lack of studies investigating the presence of these specific risk factors in the offspring of parents with versus without depression. A handful of studies have demonstrated a mediating or moderating role of coping strategies on the relationship between negative life events and child depression risk in the offspring of depressed parents [46, 50]. Nevertheless, few studies have investigated the role of multiple cognitive, affective, parenting and environmental risk factors in children of parents with depression. The current study investigates the presence of these four factors in a convenience sample of children and their parents recruited for a preventive intervention and an experimental study [51, 52]. The first aim is to replicate the finding that children of depressed versus non-depressed parents have an increased risk of psychopathology. The second aim is to determine whether various proposed mechanisms for the transmission of risk characterise children of depressed versus non-depressed parents. The final aim is to explore the extent to which these factors mediate the association between parental depression and the children’s risk of depression. Identifying the mechanisms of risk is of clinical importance since deficits in cognitive, emotion regulation and parenting skills can be targeted in preventive interventions and buffer the impact of negative life events that are highly prevalent in this high risk group [53, 54]. We hypothesize (i) that children of the HR (high-risk) group will show significantly higher psychopathology and depression symptoms, less adaptive and more maladaptive emotion regulation strategies, and a more negative attributional style compared to the children of the LR (low-risk) group. In addition, (ii) we expect that children of parents with depression perceive their parents’ parenting style as less positive and experience less positive and more negative life events. Furthermore, (iii) emotion regulation strategies, attributional style, parenting skills, and life events are expected to mediate the association between parental depression and the child’s depressive symptoms.

## Method

### Study Design

In a between-groups design, children’s psychopathology, emotion regulation strategies, attributional style, perceived parenting style and life events were compared between children of depressed (high-risk group, n = 74) versus non-depressed (low-risk group, n = 37) parents. Calculating the necessary sample size to detect group differences in potential mechanisms of transmission (emotion regulation, attributional style, stressful life events and parenting style) was difficult given the lack of studies which have directly compared these factors between children of depressed versus non-depressed parents. Assuming a medium effect size (d = 0.6), an alpha level of α = 0.05 (power 80%) and a two-tailed test the necessary sample size would be N = 90. For the mediation analysis we based the sample size calculation on an effect size of Cohen’s f^2^ = 0.40 using the same alpha and beta-levels. To address the issue of whether potentially null-effects were due to a real absence of effect versus a lack of statistical power to detect differences, we supplemented traditional hypothesis testing with Bayesian statistics.

### Participants

#### High-risk Group (HR; n = 74^2^)

Families were eligible if at least one parent fulfilled the Diagnostic and Statistical Manual of Mental Disorders DSM-IV diagnostic criteria for major depressive disorder occurring during the child’s lifetime. Parents were excluded if they suffered from alcohol/substance abuse or bipolar disorder, reported psychotic symptoms, had a personality disorder, or were in a suicidal crisis. Children and adolescents aged 8–17 years were included in the study if they did not meet the DSM-IV diagnostic criteria for a psychiatric disorder (present or past) and showed normal cognitive development (as indexed by an IQ > 85). At least one parent and one child per family were included in the study. In case both parents suffered from depression in the HR group (n = 4 families), the parent showing more severe symptoms in the clinical interview was chosen for the analysis. If multiple children within one HR family were eligible for the study (n = 53 families), the oldest child of the family (< 18 years) was selected.^3^ HR families were recruited from diverse sources but mostly in inpatient and outpatient psychiatric clinics in Munich where a parent had received treatment or by public advertisement (newspaper, radio, flyer, public transport, city council). Interested families who had heard about the prevention intervention trial were informed about the procedure by the research team and invited to the baseline assessment session if they met the inclusion criteria. Data were collected during an initial assessment session for the prevention intervention study. The HR group was a sub-set of the 100 families that were taking part in the prevention intervention study.^4^

#### Low-Risk Group (LR; n = 37)^5^

Parents and their children (aged 9–14 with an IQ > 85) were included if they did not meet the DSM-IV diagnostic criteria of any (past or present) psychiatric disorder. In order to ensure that none of the parents in the LR group had ever met criteria for a psychiatric disorder, the clinical interview was conducted with the second parent whenever possible (77% of families) and a depression screening instrument was administered (83% of families). Despite differences between the two groups in age range (HR = 8–17 vs. LR = 9–14), the mean age and standard deviation was similar for both groups (see Table 1).^6^ In the LR group, the selection of parent and child per family was random due to practicability. LR families were mostly recruited by public advertisement or via the research group’s database of former study participants and mailings of randomly picked addresses of families with children in the corresponding age range provided by the local registry office. If families were interested in the RCT trial they contacted the research team and were provided with more information about the procedure. If they met the inclusion criteria, families were invited to the department for the baseline assessment session.

**Table 1:** Demographic and behavioral characteristics, children and parents

	HR	LR	Total sample	*p*
	(n = 74)	(n = 37)	(N = 111)	
Families				
Children				
Age (M,SD)	12.0 (2.97)	11.77 (1.65)	11.92 (2.59)	n.s.
Gender (female, %)	52.7	59.3	56.3	n.s.
IQ (M,SD)	106.6 (14.76)	111.66 (11.03)	108.27 (13.77)	n.s.
School type (%)^a^				
Grundschule	28.6	31.5	37.5	n.s.
Realschule	16.6	7.9	12.5	n.s.
Gymnasium	36.4	55.3	47.1	n.s.
Parents				
Age (M,SD)	46.61 (6.33)	45.08 (4.70)	46.04 (5.86)	n.s.
Gender (female, %)	62.7	83.8	70.1	< .001
Education (%)				
Basic education	20.6	21	20.6	n.s.
A-levels	27.1	15.8	22.7	n.s.
University	42.4	57.9	48.5	n.s.
Doctoral degree	10.2	5.4	8.6	n.s.
Marital status (%)				
Married	72.6	81.1	75.5	n.s.
Separated	12.9	5.4	10.4	< .001
Employment (%)				
Full time	41.6	39.5	55.2	n.s.
Part time	22.1	60.5	40.6	n.s.
Unemployed	3.4	0	2.1	n.s.
Retired	7.8	0	6.3	n.s.
Monthly family income (%)				
< 2000 €	13.2	10.6	12.1	
2000–4000 €	39.6	23.4	33.0	
4000–5000 €	22.6	18.4	20.9	
> 5000 €	24.5	47.4	34.1	n.s.
Depressive symptoms, parent (M, SD)	17.59 (10.98)	1.79 (3.47)	12.14 (11.82)	.000

German educational system: Grundschule refers to 1st to 4th grade for all students, afterwards students seperate depending on achievement to either Mittelschule, Realschule or Gymnasium

### Procedure

Each family received €25 as reward for participating in the assessment session. All participants were informed about the study procedure and possible risks and provided written consent (parents) or assent (children under 18) for study participation. Both studies were approved by the medical faculty’s ethics committee (PRODO; Nr. 3-14, GENERAIN; 441-15) and were conducted in line with the Declaration of Helsinki.

### Measures

Table 2 gives an overview of the instruments used to determine eligibility for the study and to measure outcomes. The assessment included clinical interviews conducted by the clinically trained members of the research team (JL, KS, AS, BP, PW) or self-report questionnaires.

**Table 2:** Eligibility and outcome variables

Measure	Instrument
Eligibility criteria	
Diagnostic status (child)	K-DIPS
Intelligence (child)	CFT 20-R
Diagnostic status and history of Depression (parent)	DIPS
Personality disorder (parent)	SKID II
Psychopathology (2nd parent)	BDI-II, SCL-90-R^a^
Outcome measures	
Symptoms of depression (child)	DIKJ
Symptoms of general psychopathology (child)	YSR, CBCL
Emotion regulation strategies (child)	FEEL-KJ
Attributional style (child)	ASF-KJ
Stressful life events (child)	CASE (C/P)
Parenting style	ESI
Depressive symptoms (parent)	BDI-II
Status depression (parent)	DIPS

*K-DIPS* Diagnostisches Interview bei psychischen Störungen im Kindes- und Jugendalter, *CFT 20-R* Culture Fair Test 20. Revision, *DIPS* Diagnostisches Interview bei psychischen Störungen, *SKID II* Strukturiertes Klinisches Interview für DSM-IV, *SCL-90-R* Symptom Checklist,*DIKJ* Depressions-Inventar für Kinder und Jugendliche, *YSR* Youth Self-Report, *CBCL* Child Behaviour Checklist, *FEEL-KJ*  Fragebogen zur Erhebung der Emotionsregulation bei Kindern und Jugendlichen, *ASF-KJ* Attributionsstil-Fragebogen für Kinder und Jugendliche, *CASE* Child and Adolescent Survey of Experiences, *BDI-II* Beck’s Depression Inventory

^a^The SCL-R-20 and BDI-II for the non-affected second parent were only used as screening instruments and are therefore not included in the results

### Eligibility Measures

#### Diagnostic Status of Parents and Children

The *Diagnostisches Interview bei psychischen Störungen (DIPS)* [56] is a semi-structured, clinical interview and was used to assess whether parents met the diagnostic criteria (according to DSM-IV) for study inclusion. Previous studies report high inter-rater reliability using the instrument (κ = 0.72 and κ = 0.92) [57]. In this study, inter-rater reliability was calculated for 20% of the HR group (parents and children). These were selected randomly and re-rated by an independent researcher (LT or KM). The pre-defined criterion was the accordance of diagnosis concerning the current and previous status of depression. The accordance rate was excellent (κ = 1.00). A child version of the same instrument was used to confirm that none of the children met the criteria for a psychiatric disorder [58]. This version includes both a self- and parent-report. Where reports differed between parent and child, greater weight was given to the child-report since parents suffering from depression tend to underestimate the impact of child’s potential symptoms [59].

#### Intelligence Screening (Child)

The *Culture Fair Test* (CFT 20-R) [60] is an established non-verbal multiple-choice intelligence assessment with five response options. It is characterised by a very good re-test reliability (r = .96) and correlates well with other intelligence tests [60]. The CFT 20-R is split into two parts with four sub tests each (serial continuation series, object classification, matrices, and topologies). In this study the short-version (part one; 56 items) was used.

### Outcome Measures

#### Symptoms of Depression (Child)

We used the German version of the 26-item self-report Children’s Depression Inventory (CDI) [61], which contains statements related to symptoms of depression on a 3-point likert scale (e.g. “I enjoy most of the things I’m doing.”, “I only enjoy some things I’m doing”, and “I’m not enjoying anything at all.”) (DIKJ) [62]. The internal consistency of the DIKJ is high (Cronbach’s alpha α = 0.92) and reliability is good (re-test reliability rtt = 0.76) [62]. Construct validity is high, given that the items are directly based on the DSM-criteria for depression. External validity is moderate as evidenced by positive correlations with measures of self-esteem (r = .60) and anxiety (r = .61) [63].

#### Children’s General Psychopathology

Internalising, externalising, and general symptoms of psychopathology were assessed using self-(YSR) [64] and parent-report (CBCL) [64]. The YSR contains 123-items on a 3- to 4-point Likert scale (0 = “not at all” to 2 = “exactly true”). High internal consistencies are reported for the internal and external symptoms scale (r = .86), sufficient internal consistencies were found for other subscales (e.g. “aggressive behavior”, r > .70). The CBCL contains the same number of items and response options as the YSR. Internal consistency of the internalising and externalising subscales is high (r > .85) [64].

#### Children’s Emotion Regulation Strategies

The self-report questionnaire *Fragebogen zur Erhebung der Emotionsregulation bei Kindern und Jugendlichen* (FEEL-KJ) [65] was administered in order to evaluate how children and adolescents cope with the emotions anxiety, sadness, and anger. It consists of 90 items that assess the use of adaptive (problem focused action, distraction, increased happiness, acceptance, forgetting, cognitive reappraisal, problem solving) and maladaptive (giving up, aggressive behavior, withdrawal, negative self-evaluation, perseveration) emotion regulation strategies for each emotion. Each item (e.g. “When I’m angry, I keep my feelings to myself”) is rated on a 5-point Likert scale according to how often it is applied (“never” to “almost always”). The internal consistency is good for the subscales adaptive (α = 0.93) and maladaptive (α = 0.82) emotion regulation strategies [65]. The six-week re-test-reliability for the two subscales has been reported as good (r_tt_ = 0.81, adaptive strategies; r_tt_ = 0.73, maladaptive strategies) [65]. Sum scores for adaptive and maladaptive strategies were calculated for each emotion (anger, anxiety and sadness).

#### Child Attributional Style

The *Attributionsstil*-*Fragebogen für Kinder und Jugendliche* (ASF-KJ) [66] consists of descriptions of eight positive and eight negative situations whose causes are rated on a 4-point scale in three dimensions: internal versus external (1 = “caused by other person or circumstances” to 4 = “I caused this event”), stable vs. instable (1 = “will never be important” to 4 = “will always be very important”), and global vs. specific (1 = “is just this time relevant” to 4 = “will also be relevant at other occasions”), resulting in a total of six subscales (i.e., three dimensions for attribution of positive and three dimensions for attributions of negative situations). Coefficients of consistency (Cronbach’s alpha) of the global and stability dimension lie between α = 0.72 and α = 0.81, the internality dimension between α = 0.52 and α = 0.57 [66]. Retest-Reliability (4 weeks) was observed to vary between r_tt_ = .49 and r_tt_ = .65 and considered acceptable [66]. Since the six subscales of the ASF are each composed of only 8 items, they may indirectly affect the power of the analysis. Therefore, we used sum scores of the three dimensions of positive and negative attributional scales.

#### Parenting Style

The parenting style inventory (ESI) [67] is a 65-item child-report questionnaire in which aspects of positive (support, praise) and negative (criticism, restraint, inconsistency) parenting style are reported on a 4-point Likert scale (1 = “never or rarely happens” to 4 = “always happens”). The retest-reliability is reported to be modest (0.51–0.72), while the internal consistency of both the mother’s and father’s part is considered good (0.77–0.92 and 0.65–0.71) [67]. Sum scores for positive (praise, support) and negative parenting (criticism, restraint, inconsistency) styles were calculated.

#### Child’s Life Events

The *Child and Adolescent Survey of Experiences* (CASE) [68] is a checklist assessing the occurrence and experience of 38 life events in the last 12 months. Children (CASE-C) and parents (CASE-P) first indicate whether a specific life event happened (yes/no) and secondly how they/their child experienced this life event on a 6-point Likert scale (1 = “very pleasant” to 6 = “very unpleasant”). Scores for the number and the impact of positive as well as negative life events were calculated for self and parent report. Moderate retest-reliability (one week) for mothers and children (r_tt_ = .75) and acceptable correlations with comparable instruments (e.g. PACE, r = .28–.47) have been reported [69].

#### Parent’s Symptoms of Depression

We used the German version of the 21-item *Beck’s Depression Inventory* (BDI-II) [70] which has a 4-point Likert scale (0 = “I do not feel sad” to 3 = “I am so sad or unhappy that I can’t stand it.”). In a period of 5 months a re-test reliability of r = .78 was identified [70]. There are high correlations between the BDI-II and other questionnaires concerning symptoms of depression such as the FDD-DSM-IV (Fragebogen zur Depressionsdiagnostik nach DSM IV) [71] (r = .72–.89).

#### Missing Values

The range of missing outcome values between variables was 0.9–21.4% (x_missing_ = 13.1%), consequently above the critical value of 5% suggesting non-coincidence [71, p. 177]. Importantly, the HR and LR groups did not differ significantly in the amount of missing data (t_1,59_ = 0.23; *p* = .470) thus we assumed that data was missing completely at random (MCAR). Most missing data was observed for the ASF (21.4%), DIKJ (19.6%), YSR (18.7%), CBCL (16.1%) and FEEL-KJ (12.5%). Less missing data was observed for the CASE (0.9%) and BDI-II (2.7%). Missing values were imputed based on the expectation–maximization method [72]. This method enables imputation without changes of group means, standard deviations, and covariance.

#### Analysis Strategy

The data were analyzed using SPSS Version 19 (SPSS Inc., 1989–2006) for Windows and JASP Version 0.8.1.1 for Mac Os x for calculating additional Bayesian statistics. Differences between groups were calculated using t-tests for continuous variables and the Mann–Whitney test for dichotomous and categorical variables. Aim 1 was investigated with a two group MANOVA of symptoms of general psychopathology and depression (YSR, CBCL, DIKJ). Aim 2 was investigated with four two group MANOVAs for each of the potential mechanisms for the transmission of depression (FEEL-KJ, ASF, ESI, CASE). The mediation question (Aim 3) was investigated using a procedure that resembles the four step procedure of Baron and Kenny [73]. The first two steps coincide with our first two aims, in which we establish a relationship between the independent variable (parental depression) and the dependent variable (risk of depression in children operationalized by severity of depressive symptoms; Aim 1) and the potential mediating variables (emotion regulation; attributional style, parenting style and life events; Aim 2). The third and fourth steps are taken through hierarchical regression analyses to test the effects of the potentially mediating factors on the dependent variable, once the independent variable and any confounding factors have been added. We ran four separate regression models to test the mediating effects of the four potential mechanisms (emotion regulation, attributional style, parenting style, life events). Each mechanism was operationalized by two sub-scales: positive and negative (e.g. positive versus negative emotion regulation strategies). The first block in each stepwise regression models constituted of demographic variables of the child age, gender, IQ-score, type of school^7^ and family socio-economic status.^8^ The status of parental depression (HR, LR) was entered in a second step in all regression analyses. In the final step of each of the four regression models, the additional variance explained by the potential mechanism of transmission was tested. If the effects of the mediating variables are significant and the effects of parental depression are smaller than before then (partial) mediation is established.

Since the age range was quite large (8–17 years) and to control for gender, T-scores were used for all outcome measures that provided standard tables (DIKJ, YSR, CBCL, FEEL-KJ, ASF-KJ. ESI^9^) and additional analysis included age as a covariate to test the impact. In addition to traditional hypothesis-testing analyses, the Bayes factor (BF_10_) [75] was also calculated.

## Results

### Demographics

A total of N = 111 families were included in the study (see Table 1). The participating children were between 8 and 17 years and parents between 34 and 60 years old. In general, families had a high economic status (49.5% had a family income > 4000€ per month and 56.7% had a university degree). The majority (90.5%) of families were of German nationality, 8.5% had diverse backgrounds and acme from Bulgaria, Turkey, Italy, Austria, Brazil and Switzerland. The HR and LR group did not differ significantly in child age (t_93_ = 0.47, *p* = .639) or parent age (t_89_ = 1.63, *p* = .107), ethnicity (t_93_ = 0.90, *p* = .929), education (child: t_93_ = 0.87, *p* = .385; parent: t_91_ = − 0.35, *p* = .724), or family income (t_86_ = − 1.81, *p* = .073). Parent gender did differ between the groups (t_93_ = − 2.58, *p* < .001) with more female parents in the LR group. Children’s gender did not differ (t_93_ = − 0.76, *p* = .450). 24% of the parents were single parents (the rest in two-parent families). As expected, parents in the HR group had significantly higher BDI-II scores than those in the LR group (t_93_ = 8.72, *p* < .001). Fourty-two (71.2%) of parents in the HR group were currently depressed while 17 (28.81%) were in remission. 35% of the parents reported current comorbid disorders (25 cases of anxiety disorders and three cases of eating disorder). The mean of number of depressive episodes was 5.7 (SD = 5.5, median 3.0) with a range of only one depressive episode (10.7%) up to 20 episodes (5.8%).

### Association Between Parental and Child Psychopathology (Aim 1)

The MANOVA revealed a large and statistically significant multivariate main effect of group (HR, LR) on children’s psychopathology (Wilks’ λ = 0.565, F_1,79_ = 3.78, *p* < .001; $\eta_{\text{p}}^{2}$ = 0.435). Univariate analyses showed that for all variables (i.e. self-reported symptoms of depression and internalising, externalising and general psychopathology rated by parents and children) the HR group showed significantly higher values than the LR group (Table 3).^10^ Supporting these findings, Bayes factors indicated anecdotal (YSR; BF_10__YSR_ = 2.97) to decisive (DIKJ; BF_10 DIKJ_ = 50.01, CBCL; BF_10__CBCL_ = 27,709.01) evidence in favor of rejecting the null-hypotheses.^11^ In addition there were positive associations between the children’s and parents’ symptoms of depression (r = .23, *p* = .032).^12^ These differences were also represented by meeting clinical cut-offs (t-values > 60) in four cases of the HR compared to two in the LR on depressive symptoms, 21 (HR) versus 0 (LR) on parent-rated psychopathology and 13 (HR) versus three (LR) in the self-rated psychopathology.

**Table 3:** Group differences in children’s psychopathology

	Descriptives		Univariate effects		
	HR M (SD)	LR M (SD)	t	*df*	*p*
Self-report symptoms of depression (DIKJ)	46.79 (7.43)	40.89 (6.84)	3.62	1, 82	< .001
Youth-self report (YSR)					
Internalising symptoms	52.05 (10.35)	47.00 (8.31)	2.41	1, 92	.018
Externalising symptoms	50.86 (7.2)	46.88 (8.27)	2.41		.018
General psychopathology	53.19 (8.72)	48.70 (7.98)	2.48		.000
Child behaviour checklist, parent report (CBCL)					
Internalising symptoms	58.31 (9.53)	47.48 (6.47)	28.08	1, 89	< .001
Externalising symptoms	51.42 (7.60)	48.28 (8.01)	5.21		.025
General psychopathology	55.46 (7.73)	47.10 (7.01)	2.48		.016

*p < .05; **p < .001

### Group Differences in Potential Mechanisms for the Transmission of Depression (Aim 2)

Table 4 provides an overview of group differences in potential mechanisms for the transmission of depression in HR and LR groups.

**Table 4:** Group differences in potential mechanisms for the transmission of depression

	Descriptives		Univariate effects		
	HR M (SD)	LR M (SD)	t	*df*	*p*
Emotion regulation strategies (FEEL-KJ)					
Adaptive strategies					
Sumscore					
Anger	44.91 (12.31)	50.31 (12.29)	− 2.06		.042
Anxiety	46.18 (12.01)	51.07 (12.86)	− 2.13		.036
Sadness	48.62 (10.17)	50.13 (11.68)	− 0.90		.371
Maladaptive strategies					
Sumscore					
Anger	47.95 (10.39)	43.00 (10.51)	2.08		.040
Anxiety	46.47 (10.64)	44.34 (10.10)	0.93		.352
Sadness	45.47 (9.97)	43.65 (10.19)	1.01		.315
Attributional style (ASF-KJ)					
Positive	68.55 (9.58)	74.52 (9.79)	− 2.48	1, 86	.015
Internal	45.15 (9.04)	50.08 (10.26)	− 1.92		.018
Stable	49.96 (10.83)	56.52 (11.71)	− 1.77		.058
Global	47.92 (11.89)	53.17 (13.39)	− 0.86		.390
Negative	61.62 (10.03)	66.82 (11.40)	− 1.99		.050
Internal	44.15 (9.84)	46.73 (9.28)	− 1.21		.228
Stable	50.86 (9.88)	57.13 (10.63)	− 1.95		.056
Global	48.79 (9.84)	52.30 (13.37)	− 0.71		.481
Parenting style (ESI)					
Positive	71.63 (12.05)	80.13 (8.81)	− 3.70	1, 93	.000
Negative	50.32 (11.51)	49.90 (7.56)	1.13		.261
Rating of life events (CASE-C)					
Number of positive life events	5.38 (1.90)	6.30 (1.41)	− 3.12	1, 94	.002
Number of negative life events	3.80 (2.32)	3.20 (1.93)	0.68		.498
Impact of positive life events	13.87 (5.37)	15.12 (5.11)	− 1.56		.121
Impact of negative life events	7.80 (5.15)	6.90 (4.55)	1.36		.179

### Emotion Regulation Strategies

The MANOVA revealed statistically significant group differences (HR, LR) in children’s emotion regulation strategies of a medium-to-large effect size (Wilks’ λ = 0.872, F_1_,_91_ = 2.6, *p* = .039, $\eta_{\text{p}}^{2}$ = 0.13). Univariate tests revealed this effect to be driven by the LR group reporting more *adaptive emotion regulation strategies for anger* and *anxiety* than the HR group (Table 4). Bayes factor analysis of these subscales revealed anecdotal evidence for H0 (BF_10_ adaptive strategy anger = 0.93; BF_10_ adaptive strategy anxiety = 0.52).

### Attributional Style

The MANOVA revealed no evidence of a statistically significant multivariate main effect for group concerning children’s attributional style, (Wilks’ λ = 0.920, F_1,81_ = 1.17, *p* = .329; $\eta_{\text{p}}^{2}$ = 0.080).

### Parenting Style

The one-way MANOVA revealed a statistically significant multivariate main effect for group concerning children’s perception of their parent’s parenting style with a medium-to-large effect size (Wilks’ λ = 0.87, F_1,92_ = 6.88, *p* = .002; $\eta_{\text{p}}^{2}$ = 0.13). Univariate comparisons (see Table 4) revealed significant differences between the HR and LR groups in the positive parenting subscale (indicating a less positive parenting style perceived by the HR group than the LR group; F_1,93_ = 13.70, *p* < .001; $\eta_{\text{p}}^{2}$ = 0.13; BF_10_ = 0.48), but not in the negative parenting subscale (F_1,93_ = 1.27, *p* = .261; $\eta_{\text{p}}^{2}$ = 0.01; BF_10_ = 0.07).

### Life Events

The one-way MANOVA revealed a medium-to-large statistically significant multivariate main effect for group concerning children’s report of positive and negative life events and their rating of its impact (Wilks’ λ = 0.873, F_1,77_ = 2.8, *p* = .031; $\eta_{\text{p}}^{2}$ = 0.127). In the post hoc univariate tests (Table 4), HR children reported significantly fewer positive life events than LR children (t_94_ = − 3.12, *p* = .002), but there was no evidence of a difference between groups in the impact of positive life events (t_94_ = − 1.56, *p* = .121). There was no difference between the HR and LR children in the number of negative life events reported (t_94_ = 0.68, *p* = .498) or their impact (t_94_ = 1.36 *p* = .179). The Bayesian statistics confirmed these findings revealing strong support for the effect of number of reported positive life events (BF_10_ = 14.30), but no evidence for group differences in all other comparisons (BF_10_ number of negative life events = 0.27; BF_10_ impact of positive life events = 0.70; BF_10_ impact of negative life events = 0.58).

### Mediation Analysis (Aim 3)

Table 5 displays the results of the four multiple regression analyses in which the mediating effect of emotion regulation strategies, attributional and parenting style as well as life events on the relationship between parental depression and child risk of depression was tested.

**Table 5:** Mediating effects of mechanisms on child depression

	β	SE	Standardized β	*p*	F	df	ΔR^2^	Sig. changes in F	BF_10_
General model, Step 1 and 2									
Step 1					2.20	4, 76	.104	.077	0.41
Age	1.08	0.41	0.36	.010					
Sex	0.35	1.43	0.02	.807					
IQ	0.01	0.06	0.02	.984					
Education	− 1.12	0.71	− 0.24	.103					
Step 2					11.00	1, 75	.219	.001	6.10
Depressive status, parents	− 4.44	1.46	− 0.31	.003					
Mediating effects, Step 3									
Step 3: emotion regulation					4.89	2, 73	.311	.010	91.94
Adaptive emotion regulation	− 0.01	0.06	− 0.02	.838					
Maladaptive emotion regulation	0.22	0.07	0.31	.003					
Step 3: attributional style					0.45	2, 54	.252	.635	2.29
Positive attributional style	0.07	0.13	0.11	.587					
Negative attributional style	− 0.11	0.12	− 0.19	.362					
Step 3: parenting style					0.83	2, 72	.237	.442	4.17
Positive parenting	0.02	0.07	0.28	.808					
Negative parenting	0.08	0.06	0.14	.204					
Step 3: life events					4.01	2, 73	.296	.022	43.85
Positive life events	0.04	0.37	0.01	.908					
Negative life events	0.91	0.34	0.28	.010					

Dependent variable: child depression score (DIKJ); all variables are child variables except depressive status of parent

Beyond parental depression and the background variables, which accounted for 21.9% of variance in children’s risk of depression (*p* = .001), emotion regulation strategies significantly contributed additional variance (ΔR^2^ = 31.1%; *p* = .010). Maladaptive emotion regulation strategies had a significant beta weight (β = 0.22, *p* = .003). Since the beta weight of parental depression remained significant (β = − 4.44, *p* = .003) after including the emotion regulation strategies, we conclude that there is no *full* mediation, but that emotion regulation strategies *partially* mediate the effect of parental depression on the children’s depressive symptoms. A similar effect was found for children’s life events: After the first steps in the regression model, negative life events had a significant beta weight (β = 0.91, *p* = .010) in the regression model accounting for 29.6% in total of the variance in the dependent variable. Again, the beta weight of parental depression remained significant (β = − 4.92, *p* = .002) after including the life events. Consequently, negative life events are identified as a partial mediator in the effect of parental depression on children’s depressive symptoms. In contrast, attributional and parenting style did not significantly contribute to the variance in children’s depressive symptoms (all β weights n.s.).

## Discussion

The present study sought to investigate the factors that are associated with vulnerability for depression in the children of depressed (n = 74) versus non-depressed (n = 37) parents. This is the first study to compare the relative value of numerous risk factors in predicting risk in the offspring of depressed parents.

### Summary of Findings

Although children suffering from a psychiatric illness were explicitly excluded in this study, major differences ($\eta_{\text{p}}^{2}$ = 0.435) in sub-clinical symptoms of depression and general psychopathology between children of depressed (HR) versus non-depressed (LR) parents highlight the increased risk of depression for this group. Confirming hypothesis (i), these differences were evident across all measures of self- and parent-reported symptoms of depression and general psychopathology (internalising and externalising symptoms) and were supported by small to moderate correlations between children’s and parents’ symptoms of depression (r = .23). In addition, HR (vs. LR) children used fewer adaptive emotion regulation strategies ($\eta_{\text{p}}^{2}$ = 0.13), perceived fewer positive parenting strategies ($\eta_{\text{p}}^{2}$ = 0.13) and fewer positive life events ($\eta_{\text{p}}^{2}$ = 0.127). Interestingly, the HR children did not differ from the LR children in the maladaptive dimensions of those variables (use of maladaptive emotion regulation strategies, frequency of negative attributions, perception of negative parenting strategies, frequency and impact of negative life events). Hence, hypothesis (ii) was only met for the positive scales. Regarding the mediation analysis, maladaptive emotion regulation strategies and negative life events were identified as partial mediators in the association between parental depression and children’s depressive symptoms, confirming hypothesis (iii) partially.

### Interpretation of Findings

By demonstrating significantly more symptoms of depression and general psychopathology among the HR group with a large effect size, the data replicate earlier findings that children of depressed parents are at increased risk, not only for developing depression [5, 77], but also internalising and externalising disorders more generally [78, 79]. Participants who met diagnostic criteria for a psychiatric disorder following a clinical interview were excluded from the study. Nevertheless, more participants in the HR (versus LR) group showed values above the clinical cut-off for the depression and psychopathology outcome variables DIKJ, YSR, and CBCL. This demonstrates the clinical significance of our findings. Pending replication in longitudinal studies, the findings might be used to increase the effectiveness of interventions.

Interestingly, there was a discrepancy between the parent- and child-rated psychopathology, supporting previous studies [80, 81] with parents reporting greater symptom severity in their children than the children themselves (55.6% accordance rate). This supports Angold and Costellos [80] observation of the reduced reliability of affected parents’ ratings on children’s psychopathology, due to a loss of empathy and increased worry as a result of depression. In addition, children might spare their parents with their psychological difficulties in order to avoid burdening them even more. However, the rating of symptoms of psychopathology in the parent and child questionnaires (YSR vs. CBCL) did not differ significantly (t = 0.489, *p* = .626).

The data demonstrate support for the model of familial transmission by Goodman and Gotlib [9] which proposes risk of depression in children to be mediated by a combination of exposure to less adaptive parenting strategies, difficulties in emotion regulation, negative cognitions and increased stress exposure. HR families were characterised by a combination of less positive parenting strategies, less frequent use of adaptive emotion regulation and fewer positive life events. Although previous studies have identified these factors as playing a role in the familial transmission of psychopathology [19, 34, 82], these have largely been based on community samples or on their moderating role within samples of children of depressed parents. Few studies have directly compared these factors between children of depressed versus non-depressed parents. In this sample, the equally large effects were displayed for emotion regulation, parenting style and life events. The failure to find a contributing role for cognitive factors such as attributional style contrasts with a study by Compas et al. [19] which found that both coping strategies and a negative cognitive style contributed independently to the regression model (DV: child’s affective symptoms). We received critical feedback from participants regarding the ASF questionnaire: children said they had difficulties imagining the scenarios that they were asked to rate (i.e. they would not happen in their lives). In addition, attributional style is just one aspect of cognitive vulnerability. Other cognitive vulnerabilities include self-esteem, hopelessness and the cognitive triad, which we were not able to measure in the current study. Another limitation is that we only had scope to measure the child’s perception of the parenting style, which could be biased by social desirability. Future research concerned primarily with the effect of parental depression on parenting style should include observational and parent-reports of parenting to achieve better data triangulation.

Interestingly, although the HR group showed an absence of numerous adaptive skills (adaptive emotion regulation) and experiences (positive life events and positive parenting), there was no evidence that they showed increased maladaptive skills (maladaptive emotion regulation) and experiences (negative life events and negative parenting). These effects are contradictory to earlier findings characterising this sample with negative emotion regulation [23], negative thinking style [25], negative parenting style [37, 38] and negative life events [46, 83]. There are several possible explanations for these findings. One reason might be that the data from the high-risk sample were collected in the assessment session of an intervention study, where most parents were concerned about passing on their disorder to their children. Consequently, these parents may have been reflecting on their behavior and trying to protect their children from negative influences (e.g. negative thinking and behavior). In addition, the majority of parents of the HR group (92.4%) had received psychological treatment for depression before entering the study and might therefore be better informed about maladaptive coping strategies and parenting than parents (in the LR group) who had not received psychotherapy.. Nevertheless, children might lack a role model who is displaying adaptive coping strategies. In this sample, children of depressed parents differed significantly from children of non-depressed parents in their report of positive (but not negative) life events. Fewer positive life events might indirectly mirror the environment that goes along with parental depression (e.g. less family activities, holidays) as well as reflecting more indirectly—similarly to other psychological skill deficits—financial problems and conflicts in marriage that go along with parental depression. Since parents with depression experience more stress (e.g., financial problems, unemployment, etc.), we expected their children to report more negative life events (e.g., divorce of parents) [46, 83]. However, this was not the case. One reason for this might be that the assessment was based on children’s reports of life events and children were not aware of negative life events (e.g., financial, marital problems of parents) since parents might try to shield negative life events from their children in order to protect them. The fact that the sample had a high socio-economic background may also be an important factor in explaining why children reported fewer difficulties. In addition, we mainly assessed the mechanism variables via self-report measures and desirability biases may have played a role. Future assessment and interventions with this population should consider the fact that they may not only suffer from negative life events but also from a lack of positive life events.

This is the first study to examine the extent to which several potential mechanisms may account for the relationship between parental depression and child psychopathology. Although numerous factors characterised the children of depressed versus non-depressed parents (emotion regulation, attributional style, life events, parental depression), not all factors contributed significantly in the regression model which sought to account for variance in children’s own psychopathology. Both maladaptive emotion regulation strategies and negative life events (but not attributional and parenting style), significantly predicted the children’s symptoms of depression above the background variables and parental depression. If these findings are replicated in experimental studies, prevention interventions which focus on reducing the use of maladaptive emotion regulation strategies in the face of negative life events may be particularly effective.

### Strengths and Weakness

The main strength of this study is the direct comparison of multiple risk factors in a sample of children of clinically depressed parents with children of non-affected parents which enables stronger conclusions about the trans-generational effect of parental depression than studies based on community samples. Although a larger sample size would have enabled us to calculate more robust statistical models e.g. structural equational modally, recruiting families with a mentally ill parent is particularly time consuming [84] and the power was sufficient to calculate between-group differences in potential mechanisms. Furthermore, we conducted Bayes analyses to test the extent to which any null-effects were due to a lack of statistical power.

A number of clinically-relevant instruments were used to characterize the sample. Clinical interviews (with high interrater-reliability; κ = 1) were conducted with both parents and children. As such, we could be sure about the diagnostic status of parents in both groups. This also enabled us to exclude children who had a lifetime diagnosis of a psychiatric disorder and be more certain that any vulnerabilities identified were antecedents, not consequences, of depression. On top of that, child- and parent-ratings of symptoms of general psychopathology were included. Parent-ratings alone have been shown to be less valid for children’s internalising symptoms but more valid for externalising symptoms [85, 86]. Including both ensures a more comprehensive interpretation of the data.

One limitation of the study is the representativeness of the sample. The HR group was an opportunistic sample of families who were recruited for an intervention whereas data of the LR group were collected within an experimental study. The opportunity to receive and intervention may have meant that the HR group were generally more motivated to participate than the LR group, although in this case any group differences in terms of risk of depression are likely to *underrepresent* the true difference in the general population. Although there were differences in marital status and gender between the HR and LR groups, there was no evidence that these differences affected the findings. Since taking part in a time-consuming intervention (12sessions in within 6 months) with the whole family demands a high level of motivation, especially for depressed parents, the investigated sample might not be representative of children of depressed parents in general. Furthermore, the socio-economic background of all families was high and rarely had a migration background^13^; nevertheless, they differed in parent gender and marital status making the groups less comparable. In addition, few parents suffered from severe depressive episode in the sample and showed great variability in their course of depression. Following this argumentation, the effects we found might be even stronger in the general population of families with a depressed parent, since the families with more risk factors due to their socio-economic background were underrepresented in our sample. Nevertheless, our sample might be quite informative for future interventions for children of depressed parents, since it represents a subgroup that enrols in such interventions.

Although missing values could be imputed, a limitation of this study is the amount of missing data. Since this study is about families with parental depression, it is not surprising that impairments of those families are mirrored in the response rates of questionnaires. Although families were encouraged to fill in the questionnaires properly and reminded to send them back soon, many parents felt stressed by just another “to do” in their daily routine.

We included the factors in the Goodman and Gotlib [9] model that we believed to be most important in the transgenerational transmission of depression but could not include other factors such as parental cognitive style and affect, which via social learning are proposed to explain the cognitive and affective vulnerabilities in offspring. Future research focusing on *how* offspring of depressed parents acquire negative cognitive and affective vulnerabilities could also inform preventive interventions.

A final limitation of the study is that the data is cross-sectional rather than longitudinal and therefore does not allow causal interpretations to be drawn about the factors which prospectively predict the onset of a depressive episode in the children of depressed parents. In order to capture developmental risks and model resilience for depression, longitudinal studies are needed. Furthermore, additional information regarding familial factors as structure, cohesion and parent–child interaction is needed.

### Future Research

There are numerous avenues for future research. Although the present study combines several relevant risk factors in order to understand the transmission of depression, the data is rather exploratory. Longitudinal studies investigating representative and larger samples are needed to explore the role of risk and protective factors that were found to characterise children of depressed parents. In addition, more characteristics of the parental depression such as age of onset as well as parental cognitive style and affect (as discussed by Goodman and Gotlib, 49), and its impact on the development of a “depressotypic-style” in children are worth investigating [7]. Furthermore, data that allows structural equation modelling in order to achieve a better understanding of impact, overlap, and interaction of risk factors including gene-environment interactions [87] would be beneficial. Furthermore, it remains unclear whether the included risk factors have moderating or mediating effects. As inconsistent and unclear findings of the mediating and moderating roles of those risk factors have been discussed in numerous prior studies [7, 44, 53], future research ought to investigate these specific mechanisms in order to better understand the transgenerational pathways of depression. Moreover, additional instruments for the assessment of the cognitive factors (e.g. self-esteem, self-efficacy) may be beneficial. Further experimental data for verifying the effects of risk factors on the children’s wellbeing might also be helpful for a better understanding of transmission of depression in this high-risk group.

### Summary

Children of depressed parents are at heightened risk for developing depression, yet relatively little is known about the specific mechanisms responsible. This study compared various potential mechanisms in children of depressed versus non-depressed parents and explore mediators between parental depression and risk in offspring Children of the HR group in comparison to the LR group showed significantly more symptoms of depression and general psychopathology, less adaptive emotion regulation strategies, fewer positive life events and fewer positive parenting strategies. Maladaptive emotion regulation strategies and negative life events were identified as partial mediators of the association between parental depression and children’s risk of depression. The study confirms the elevated risk of depression in children of depressed parents and provides empirical support for existing models of the mechanisms underlying transmission. Interestingly, the high-risk group was characterised by a lack of protective rather than increased vulnerability factors. Since preventive interventions for children of depressed parents group show small effects which diminish over time [88], it is crucial to uncover the key risk factors for depression in this high-risk population. In addition to exploring the effects of existing interventions on these risk factors, future research should target these risk mechanisms specifically in preventive interventions in order to improve their efficacy.

## Electronic supplementary material

Below is the link to the electronic supplementary material.

Supplementary material 1 (DOCX 18 kb)

## Footnotes

1 *Note* Distraction is regarded as adaptive secondary coping strategy for stressors that can’t be changed by active behavior like parental depression.

2 The HR group was recruited as part of a randomised controlled trial of a family-based preventive intervention in a German city [51].

3 Since depressive symptoms increase with age [55], we chose the oldest child in order to have a greater chance of variability of symptoms.

4 The intervention was a family- and group-based program based in principles of cognitive behavioural therapy including 8 weekly and 4 monthly booster sessions. The main aim of the intervention is to increase child by teaching coping strategies, parenting skills and enhance positive family interaction.

5 The LR group were recruited from a transgenerational study of cognitive biases (study protocol described elsewhere).

6 Results were double checked for age discrepancies by conducting the analyses with only 9–14 years old children of the HR group, but patterns of results did not change.

7 German school system: Elementary school = Grundschule 1st to 4th grade, then depending on achievement (decreasing): Gymnasium 5th to 13th grade, Realschule 5th to 10th grade, Mittelschule 5th to 9th grade.

8 Socio economic status defined based on the Winkler index (income, education, economic position) [74].

9 For ESI, no standard tables were available, so sumscores of raw values were calculated.

10 Since the groups differed in parental marital status (p = < .001) and parent gender (p = .001), this variable was included as a covariate in each analysis. However, since none of the findings changed when this variable was included, results are reported for the analysis without the variable included. Parent gender also differed between the groups but since this simply referred to the parent who attended the experimental session in the LR group (and therefore had no real meaning) this variable was not included in the analyses.

11 Interpretations of Bayes Factor [76].

12 Correlations between depressive symptoms of children and parents within groups: HR: r = − .009, *p *= .953; LR: r = − .176, *p *= .234; see Supplement 1 for a correlation matrix of all factors.

13 Children whose parents were not born in Germany.
