# Supplementary material for: Risk of Depression in the Offspring of Parents with Depression: The Role of Emotion Regulation, Cognitive Style, Parenting and Life Events
Source: Child Psychiatry Hum Dev. 2019 Nov 5;51(2):294–309. doi: 10.1007/s10578-019-00930-4 (PMC7067707; doi:10.1007/s10578-019-00930-4)
Supplement: Supplementary file 1 — Supplementary material 1 (DOCX 18 kb) [file 10578_2019_930_MOESM1_ESM.docx]

|  | AS | MS | PAS | NAS | PP | NP | PLE | NLE | DS | BDI-II |
| --- | --- | --- | --- | --- | --- | --- | --- | --- | --- | --- |
| Depressive symptoms. child (DIKJ) | -.14 | .24^**^ | -.13 | -.07 | -.21^**^ | .08 | -.09 | .18^*^ | .36^**^ | .23^**^ |
| Adaptive emotion regulation strategies (AS. FEEL-KJ) |  | -.05 | .13 | .03 | .19^**^ | .00 | .01 | -.06 | -.14 | -.09 |
| Maladaptive emotion regulation strategies (MS. FEEL-KJ) |  |  | -.05 | .00 | -.07 | .10 | -.01 | .17^*^ | .10 | .01 |
| Positive attributional style (PAS. ASF) |  |  |  | .56^**^ | .21^**^ | .05 | .20^*^ | .10 | -.29^**^ | -.20^*^ |
| Negative attributional style (NAS. ASF) |  |  |  |  | .16 | -.01 | .19^*^ | .16 | -.21^*^ | -.16^*^ |
| Positive parenting (PP-ESI) |  |  |  |  |  | .08 | .15* | -.03 | -.23^**^ | -.16^*^ |
| Negative parenting (NP-ESI) |  |  |  |  |  |  | .01 | .10 | .03 | -.01 |
| Positive life events child (PLE-CASE) |  |  |  |  |  |  |  | .16 | -.31^**^ | -.19^**^ |
| Negative life events child (NLE-CASE) |  |  |  |  |  |  |  |  | .02 | .12 |
| Depressive status^1^ (DS) |  |  |  |  |  |  |  |  |  | .63^**^ |
| Depressive symptoms. parent (BDI-II) |  |  |  |  |  |  |  |  |  |  |
| *Note*. All variables are child outcome variables except the parental depressive status (DS) and symptoms (BDI-II), DS codes as 1= never depressed, 2 = remitted, 3 = currently depressed | | | | |  |  |  |  |  |  |

Supplement 1 Correlation matrix (Pearson's r) of parent and child outcome variables
